# Interactive pre-surgical design of optimal extra-cardiac Fontan connections starting with cardiac MRI reconstructions of the Glenn anastomosis

**DOI:** 10.1186/1532-429X-17-S1-P197

**Published:** 2015-02-03

**Authors:** Prahlad G Menon, Haifa Hong

**Affiliations:** Electrical and Computer Engineering, Sun Yat-sen University - Carnegie Mellon University Joint Institute of Engineering, Pittsburgh, PA USA; Bioengineering, University of Pittsburgh, Pittsbugh, PA USA; Shanghai Children’s Medical Center, Shanghai, China

## Background

Pre-surgical planning for Fontan procedures involves time-consuming patient-specific vascular segmentation and non-intuitive virtual surgical modeling which has encumbered utility in the clinic by surgeons. We present an interactive platform to design virtual surgical options and visualize pericardial enlargement strategy for Fontan connections, starting with patient-specific vascular templates.

## Methods

**Figure 1:**
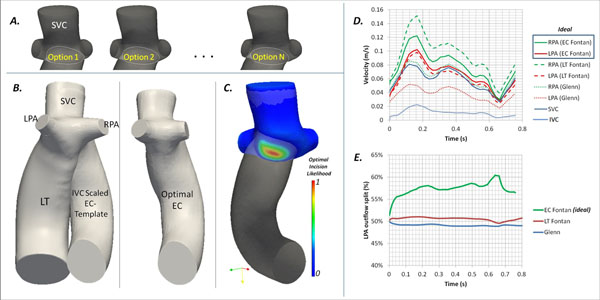
**(A) The series of experimental virtual EC Fontan connection options ranging from options with the EC conduit offset toward the LPA (in Option #1) to toward the RPA (in Option N).** (B) The patient-specific 3D LT Fontan reconstruction depicted along with a positioned and scaled EC Fontan template matching the hydraulic diameter of the patient-specific IVC. C) The ideal EC Fontan configuration which resulted in 38% reduction in mean pressure gradient from the IVC to PAs, superimposed with a colored model of the virtual pre-Fontan Glenn anastomsis depicting a likelihood (min: 0 to max:1) estimated by a regional distance mapping function to an guide optimal incision which will result in this ideal EC Fontan configuration. (D) illustrates flow velocity vs. time plots generated from CFD in the patient-specific LT case, the virtual pre-operative Glenn configuration the ideal EC Fontan construction, and (E) shows the corresponding time-varying LPA outflow splits over time for each case.

We demonstrate the utility of our novel virtual surgical construction tool in a patient-specific case of retrospectively identifying an optimal extra-cardiac (EC) Fontan connection strategy starting with post-operative cine cardiac MRI scans of a pediatric patient with a Lateral Tunnel (LT) Fontan connection. First, a 3D vascular model of the LT Fontan (including inferior & superior vena-cavae (IVC, SVC), left & right pulmonary arteries (LPA, RPA)) was segmented at end-diastole (in ITK-SNAP). A virtual pre-Fontan Glenn vascular model was derived from this LT model by virtually clipping the LT connection away using Paraview (Kitware Inc.) and fitting a smooth surface to the remaining geometry using a Poisson Surface Reconstruction (PSR) algorithm. A series of experimental EC Fontan options were interactively prepared by employing PSR to stitch together the pre-operative Glenn model with an EC conduit template in a range of feasible positions (see Figure 1A), smoothing the anastomosis junction to simulate pericardial enlargement. The template EC conduit was scaled to match the hydraulic diameter of the IVC of the patient and curved toward the LPA. Computational fluid dynamics (CFD) analysis was performed on each model (using OpenFOAM) to identify the EC model with the most favorable distribution of caval flow to the PAs and a maximum reduction in pressure gradient between IVC and PAs relative to the original LT connection. Pulsatile flow was modeled based on phase-contrast MRI caval inflow wavefoms and zero pressure-gradient outlet boundary conditions at the PAs (initialized to LPA:RPA::50:50).

## Results

The ideal tested EC connection anastomosis was offset toward the RPA (see Figure 1B,C) and resulted in a favorable 58% LPA flow split, as opposed to an equitable (~50:50, unfavorable) outflow split between LPA & RPA in the LT case which is known to correlate with increased energy loss due to collision of caval inflow streams. Figures 1D & E compare the inflow / outflow waveforms and the LPA outflow split % in the ideal EC case, with the original LT and the virtual Glenn model. The ideal EC connection had a 38% lower average pressure gradient between IVC and PAs, compared with the original LT connection.

## Conclusions

We demonstrate the feasibility of a novel virtual surgery tool to retrospectively create and hemodynamically evaluate a range of feasible patient-specific EC Fontan connections starting with a virtual Glenn anatomosis model. This presented methodology may be applied to prospective surgical planning starting with pre-Fontan CMR imaging of the Glenn anatomosis.

## Funding

N/A.

